# The first report on the prevalence of soil-transmitted helminth infections and associated risk factors among traditional pig farmers in Bali Province, Indonesia

**DOI:** 10.14202/vetworld.2022.1154-1162

**Published:** 2022-05-10

**Authors:** Kadek Karang Agustina, I. Made Ady Wirawan, I. Made Sudarmaja, Made Subrata, Nyoman Sadra Dharmawan

**Affiliations:** 1 Department of Veterinary Public Health, Faculty of Veterinary Medicine, Udayana University, Denpasar 80225, Bali, Indonesia; 2 Post-Graduation Program, Faculty of Medicine, Udayana University, Denpasar 80225, Bali, Indonesia; 3 Department of Public Health and Preventive Medicine, Faculty of Medicine, Udayana University, Denpasar 80225, Bali, Indonesia; 4 Department of Parasitology, Faculty of Medicine, Udayana University, Denpasar 80225, Bali, Indonesia; 5 Department of Veterinary Clinical Pathology, Faculty of Veterinary Medicine Udayana University, Denpasar 80225, Bali, Indonesia

**Keywords:** *Ascaris*, hookworm, risk factor, soil-transmitted helminthiasis, traditional pig farmer, *Trichuris*

## Abstract

**Background and Aim:**

Pigs are the main livestock commodity in Bali Province, Indonesia, where traditional farming practices are widely used. Traditional pig farmers are often closely associated with poverty and a perceived lack of knowledge regarding health and hygiene. Data on soil-transmitted helminthiasis (STH) and risk factors associated with STH worm infection among traditional pig farmers in Bali were previously unavailable. This study aimed to analyze the prevalence and risk factors for STH infections among traditional pig farmers in Bali Province, Indonesia.

**Materials and Methods:**

This study involved the fecal examination of 238 traditional pig farmers from all areas of Bali Province in Indonesia. In addition, several pig feces samples were combined into one pooled sample belonging to each farm. All fresh fecal samples were stored in a 5% formaldehyde solution before being analyzed using concentration flotation techniques. Subsequently, risk factors were determined through an interview and a questionnaire. The odds ratio (OR) and Chi-square tests were used to determine the risk factors associated with STH infections.

**Results:**

The result showed that there was a high prevalence of STH infections among traditional pig farmers and pig farms in Bali, with rates of 21.8% and 76.5%, respectively. This could be due to risk factors, such as personal hygiene (OR: 5.756; confidence interval [CI]: 2.96-11.193; p=0.00), sanitation (OR: 1.914; CI: 1.024-3.576; p=0.042), education level (OR: 7.579; CI: 2.621-21.915; p=0.00), household income (OR: 2.447; CI: 1.122-5.338; p=0.025), and occupation (OR: 2.95; CI: 1.356-6.415; p=0.006).

**Conclusion:**

The infections seen in farmers were distributed among hookworm, *Ascaris* spp., and *Trichuris* spp., at 15.1%, 9.2%, and 4.2%, respectively. The risk factors associated with infections of STH and *Ascaris* spp. were personal hygiene, home sanitation, education level, household income, and having a primary occupation as a traditional pig farmer. In contrast, personal hygiene, education level, and primary occupation were the only risk factors for hookworm infection, while personal hygiene and home sanitation were the risk factors associated with *Trichuris* spp. infection. The limitation of this study was that the number of samples was relatively small due to the difficulty of obtaining stool samples from traditional pig farmers, with many individuals refusing to provide their stool for inspection. We suggest that future research focus on identifying the species of worms that infect traditional pig farmers and to better identify the zoonotic link of STH transmission from pigs to humans.

## Introduction

The prevalence of helminth infections is still high in developing countries [1], which could result from a lack of concern in affected communities regarding these diseases [2]. Although these diseases do not cause sudden outbreaks or severe casualties, they slowly erode human health by causing permanent disability, impairing children’s intelligence, and in some cases leading to death [3,4]. Furthermore, the high prevalence of these diseases in certain countries may be due to low socioeconomic status, unhygienic living conditions, and a scarcity of safe drinking water among those living in endemic areas [5,6]. In addition, the disease is usually latent and poorly monitored by health-care professionals [7].

The term “soil-transmitted helminth/helminthiasis” (STH) refers to a group of parasitic diseases caused by nematode worms that spread through feces-contaminated soil [8]. The STH of major importance to humans are *Ascaris* spp., *Trichuris* spp., and hookworm. According to the most recent estimates, over 2 billion people are infected with these parasites [9].

Worm diseases caused by STH continue to be a significant public health problem in Indonesia, especially in rural areas [10,11]. The diseases primarily affect children, with a prevalence of 28.12% [12]. STH infections are known to impair patients’ health, nutrition, intelligence, and productivity owing to the loss of carbohydrates, proteins, and blood. This then leads to economic losses and the deterioration of the quality of human resources [13].

Previously, among zoonotic STH *Ascaris* spp. worms, the only known *Ascaris* sp. was *Ascaris* *lumbricoides*, but more recently, *Ascaris suum* was discovered, which is also capable of infecting humans [14-17]. A study on school children in Bali found a high prevalence of ascariasis, which was 73.7% [18]. The high prevalence of ascariasis among children is strongly associated with hygiene and frequent contact with the soil. In addition, it has also been established that *A. suum* is a zoonotic agent derived from pigs in non-endemic areas, such as the United States, Denmark, and Japan [16,17,19].

Hookworm disease is caused by *Necator americanus* and *Ancylostoma duodenale*, with *N. americanus* being the primary pathogen [20]. The disease is a public health issue due to its significant effect on the health and socioeconomic well-being of individuals in developing countries [21]. In addition, hookworm infection is associated with poverty, as well as the ability to maintain proper personal hygiene and sanitation [22,23]. Certain other hookworm species are zoonotic [24], such as *Ancylostoma* *ceylanicum* and *Ancylostoma* *caninum*, which have been occasionally reported in humans [25]. *Trichuris trichuria* infection is another public health problem [26] due to its symptoms, such as diarrhea, malnutrition, growth retardation, and anemia, which can ultimately be fatal. However, acute infections of *T. trichuria* are mostly asymptomatic [27]. According to a recent study, *T. trichuria* causes approximately 465 million infections worldwide [28].

Helminthiasis prevalence is affected by several factors in Indonesia, such as climate, personal hygiene, sanitation, environment, and population density [29]. Worm disease in patients is also frequently associated with environmental conditions, socioeconomic status, and level of educational attainment [30]. Bali Province is a small (5,700 km^2^) island in Indonesia with a dense population (4.32 million people). It has a few urban areas and some suburban areas [31]. The Balinese have a diverse range of occupations, from farmers to office workers [32]. Hinduism is the majority religion in the community, with pork being consumed and used in almost every ceremony. Pigs are raised by almost every household in rural areas [33]. In general, the animal and livestock husbandry systems in Bali follow a traditional pattern [33-37], which provides a significant opportunity for the transmission of zoonotic diseases [38]. In addition, it has been proposed that poor maintenance systems can foster the development of disease agents, especially parasites [39-41]. However, there are currently no reports of STH infection among the traditional pig farmers in Bali.

This study aimed to estimate the prevalence of STH worm infection and analyze the associated risk factors among traditional pig farmers in Bali, Indonesia.

## Materials and Methods

### Ethical approval

This study was approved by the Ethics Committees in Udayana University, with Ref. No. B/223/UN14.2.9/PT.01.04/2020.

### Study design, period, and area

This was a cross-sectional study conducted between January 2020 and October 2021 in the Indonesian province of Bali. A total of 238 samples were collected consecutively from traditional pig farmers. The inclusion criteria were pig farmers who practiced pig farming using a traditional pattern.

### Data collection and laboratory analysis

The data collection method used in this study was observational, and in-depth interviews were conducted with each traditional pig farmer using a questionnaire guide. This was developed in accordance with the previous studies [7,42-46]. The recorded risk factors include personal hygiene, sanitation, medical treatment history, education level, household income, primary occupation (i.e., a traditional pig farmer), presence of STH-infected pigs, and the presence of a pigsty in the yard.

Fresh fecal samples from farmers and pigs were collected from all regencies in Bali Province. Bali Province has eight regencies and one city, and each district and city sampled 25-30 farmers. For samples of pig feces, several stool samples were grouped into a single sample belonging to each farm. All samples were preserved in a 5% formaldehyde solution at room temperature and later examined in the Parasitology Laboratory, Faculty of Veterinary Medicine, Udayana University [41]. Concentration flotation techniques were used to examine the feces to detect helminth eggs. Approximately 10-20 g of feces was dissolved in 10-12 mL of saturated saline solution. Filtration was used to separate dregs and large dirt particles. Centrifugation was conducted for 5 min at 252 x g. The sample tubes were placed in an upright position, and then saturated salt solution was added until the tube was full and the surface of the liquid looked convex. The prepared tubes were allowed to stand for 2-3 min so that the worm eggs would float to the surface. Samples were transferred to glass slides, cover glass was placed over the surface of the liquid, and they were observed with a microscope (Olympus CX21, Japan) under 100× magnification [47,48].

### Statistical analysis

Data were analyzed using Statistical Package for the Social Sciences (IBM Corp., NY, USA). The data were tested for normality using the Kolmogorov-Smirnov test, p>0.05, and nominal variables are described in frequency and proportion. Bivariate analyses were conducted using the Chi-square test to obtain the association between the variables that were risk factors for STH infection, and the odds ratio (OR), 95% confidence interval (CI), and p*-*value were also calculated [42].

## Results and Discussion

Soil is important in the life cycle of STH because it provides a warm, moist environment for eggs to incubate and become infectious [49]. Hookworm eggs require up to 14 days to become viable and infectious, *A. lumbricoides* eggs require between 8 and 37 days, and *T. trichiura* eggs require between 20 and 100 days [50]. Infectious eggs of *Ascaris* and *Trichuris* can survive for a few months, while hookworm larvae can survive for a few weeks. Two genera of STH, namely, *Ascaris* spp. and *Trichuris* spp., are transmitted through the ingestion of infectious eggs. In addition, the larvae of hookworm species, such as *A. duodenale* and *N. americanus*, infect humans by penetrating the skin, while *A. duodenale* and *A. ceylanicum* are transmitted through the consumption of infectious larvae [51]. Helminth eggs transmitted through the soil are excreted in the feces of infected people and animals and are then present in the environment of endemic areas [49]. STH infection is a chronic infection that is a major health problem throughout the world, especially in developing countries [52]. STH infection is a neglected tropical disease and a significant health problem in Indonesia, especially in rural areas [11].

STH infections were prevalent in 21.8% of traditional pig farmers evaluated, while 76.5% of pig farms were found to be infected (Tables-1 and 2). A total of 52 farmers with confirmed STH infections were treated with Albendazole at the recommended dosage. The proportion of STH infections detected during stool examination is shown in Table-1, with infections caused by *Ascaris* spp., hookworm, and *Trichuris* spp. accounting for 9.2%, 15.1%, and 4.2%, respectively.

**Table 1:** STH infection in traditional pig farmers in Bali.

Worm	Infection	Prevalence
STH	52	21.8%
*Ascaris* spp.	22	9.2%
Hookworm	36	15.1%
*Trichuris* spp.	10	4.2%
Combination of STH infection:		
*Ascaris* spp. and hookworm	9	3.78%
*Ascaris* spp. and *Trichuris* spp.	1	0.42%
Hookworm and *Trichuris* spp.	5	2.1%
*Ascaris* spp., hookworm, and *Trichuris* spp.	0	0%

STH=Soil-transmitted helminthiasis

**Table 2:** STH infection in the traditional pig farm.

Worm	Infection	Prevalence
STH	182	76.5%
*Ascaris* spp.	87	36.6%
Hookworm	163	68.5%
*Trichuris* spp.	11	4.6%
Combination of STH infection:		
*Ascaris* spp. and hookworm	71	29.8%
*Ascaris* spp. and *Trichuris* spp.	5	2.1%
Hookworm and *Trichuris* spp.	5	2.1%
*Ascaris* spp., hookworm, and *Trichuris* spp.	2	0.84%

STH=Soil-transmitted helminthiasis

This study is the first report that shows the prevalence of STH infection among traditional pig farmers in Bali. A higher prevalence of STH infection was found in this population compared with a previous cross-sectional study that found a prevalence rate of 5.1% among vegetable farmers in Semarang City [53]. However, this study was consistent with several other studies that show an overall high prevalence of STH infections. According to a study conducted in the Coastal Savannah zone of Ghana, the prevalence of STH infections among livestock farmers was 20% [54]. Similarly, it was discovered in another study that 13.58% of vegetable farmers in Klungkung District in Bali were infected with STH [55], which was lower than the 59.9% reported by another study conducted among Orang Asli subgroups in Peninsular Malaysia [56].

This study also discovered that in Bali, hookworm infection was the most prevalent type of STH infection, followed by *Ascaris* spp. and *Trichuris* spp. infections. It is worth noting that the most common hookworm species that infect humans include *A. duodenale* and *N. americanus* [24], while another hookworm species that has been reported to cause infection in humans is *A. ceylanicum*, also known as the dog and cat hookworm [57]. Hookworm is a fatal infection, though it is generally considered asymptomatic due to its “silent and insidious” nature [58]. The prevalence of hookworm was found to be significantly higher in this study than in previous reports examining vegetable farmers in Ghana and Klungkung District, at 4.72% and 0.61%, respectively [55]. According to Ngui *et al*. [56], in a cross-sectional study, the prevalence of hookworm infection among Orang Asli subgroups in Peninsular Malaysia was 9.1%. The prevalence of *Ascaris* spp. in this study was 9.2%, with *A. lumbricoides* being the most common parasite seen [59,60]. Other previous research discovered that *A. suum* also caused infection in humans [14-17], but at a lower prevalence than what was seen in vegetable farmers in Ghana and Peninsular Malaysia, where 15.77% and 26.7% of farmers were infected, respectively [56,61]. Meanwhile, there was a low prevalence of *Ascaris* spp. infection among vegetable farmers at Klungkung District in Bali, with 1.85% being infected [55], while *T. trichiura* remained the cause of trichuriasis [62,63]. Furthermore, these parasites are a major source of infection for humans in Asia and Africa due to inadequate sanitation and hygiene standards [43]. The prevalence of *Trichuris* spp. infection in Orang Asli subgroups of Peninsular Malaysia and vegetable farmers in Klungkung District was 54.3% and 9.26%, respectively [55,56].

The most common infection finding in this study was singular infections, although the presence of some STH coinfection was observed. This involved the combination of *Ascaris* spp. and hookworm in nine cases, hookworm and *Trichuris* spp. in five cases, and *Ascaris* spp. and *Trichuris* spp. in one case. However, coinfection with all STH was not observed in any host. These results showed a lower coinfection prevalence compared with previous studies in Cameroon, where the prevalence of coinfection was 26.7% [64], and in Kenya, where the prevalence of *Ascaris-Trichuris* coinfection in school-aged students was 2.9% [65].

Other results have also shown a high prevalence of STH infections among traditional pig farmers, with 76.5% being infected, as shown in Table-2. The most common infection was the hookworm infection, followed by infections caused by *Ascaris* spp. and *Trichuris* spp. at 68.5%, 36.6%, and 4.6%, respectively. The STH coinfection discovered in this study involved coinfections of *Ascaris* spp. and hookworm, *Ascaris* spp. and *Trichuris* spp., and hookworm and *Trichuris* spp. at a prevalence of 29.8%, 2.1%, and 2.1%, respectively. Furthermore, the prevalence of all STH coinfections on the same farm had a prevalence of 0.84%, which was higher than what was observed in a previous study involving a lowland pig farm in Bali, which found an 18% prevalence of *Ascaris* spp. [66]. Meanwhile, the prevalence of hookworm was consistent with another study [67], where the prevalence was found to be 71% in lowland pig farms and 60% in Bali Province [37]. Another study identified *Oesophagostomum dentatum* and *Hyostrongylus* *rubidus* as the hookworm species that infected pigs in Bali [37]. The prevalence of *Trichuris* spp. infection in this study was consistent with a previous report that discovered a prevalence of 14% in piglets sold at the traditional market in Bali [36].

The prevalence of STH infections was found to be closely associated with environmental and socioeconomic factors [29]. Subsequently, the geospatial distribution of STH was found to be influenced by several factors, such as poor environmental sanitation, a lack of personal hygiene, the use of contaminated water, and other factors including age, socioeconomic status, and occupation [52]. This was a novel study based on the risk factors for STH infections among traditional pig farmers in Indonesia. These were discovered to be personal hygiene, home sanitation, education level, household income, and primary occupation (Table-3).

**Table 3:** Risk factor of STH infection in traditional pig farmers in Bali.

Risk factor	Positive		Negative		OR (95% CI)	p-value
	
	Frek	%	Frek	%		
Personal hygiene					5.756 (2.96-11.193)	0.000**
Good	17	7.14	137	57.56		
Poor	35	14.7	49	20.59		
Sanitation					1.914 (1.024-3.576)	0.042*
Good	21	8.8	105	44.1		
Poor	31	13	81	34		
Medical control					1.186 (0.531-2.65)	0.677
Good	9	3.78	37	15.55		
Poor	43	18.07	149	62.6		
Education level					7.579 (2.621-21.915)	0.000**
High	4	1.68	72	30.25		
Low	48	20.17	114	47.9		
Income rate					2.447 (1.122-5.338)	0.025*
High	9	3.78	63	26.47		
Low	43	18.07	123	51.68		
Main job as a pig farmer					2.95 (1.356-6.415)	0.006**
No	9	3.78	71	29.83		
Yes	43	18.07	115	48.32		
Presence of STH infected pigs					1.913 (0.841-4.351)	0.122
No	8	3.36	48	20.17		
Yes	44	18.49	138	57.98		
Presence of pigsty in yard					1.303 (0.684-2.483)	0.421
No	33	13.87	129	45.2		
Yes	19	7.98	57	23.95		

** Highly significant,

* Significant, OR=Odds ratio, CI=Confident interval

The personal hygiene activities and practices of traditional pig farmers in this study included frequent contact with soil, washing their hands with soap before meals and after coming into contact with soil or livestock, washing their hands with soap after bowel movements, habits related to wearing footwear, and unhygienic practices. The results showed that farmers and families with poor personal hygiene were 5.756 times more likely to contract STH infections than those with good personal hygiene (OR: 5.756; CI: 2.96-11.193; p=0.00). In addition, personal hygiene was a risk factor for infection with *Ascaris* spp. (OR: 7.561; CI: 2.678-21.349; p=0.00) (Table-4), hookworm (OR: 3.548; CI: 1.704-7.391; p=0.001) (Table-5), and *Trichuris* spp. (OR: 8; CI: 1.658-38.598; p=0.01) (Table-6). Previous studies have found that there was a significant relationship between handwashing with soap before meals and the incidence of worm infection [68,69]. Hand washing is a personal hygiene habit that involves cleaning the hands and fingers with soapy water or other liquids. According to the World Health Organization, handwashing is an important hygiene practice for disease prevention since it quickly and effectively impedes pathogen transmission [70]. Other significant risk factors for STH infection involved unhygienic practices, such as open defecation, untrimmed nails, and eating food that has fallen to the ground [7]. Another study found that the two most significant risk factors for STH infections in Ethiopian schoolchildren were untrimmed nails and not washing hands before meals [52].

**Table 4:** Risk factor of *Ascaris* spp. infection in traditional pig farmers in Bali.

Risk factor	Positive		Negative		OR (95% CI)	p-value
	
	Frek	%	Frek	%		
Personal hygiene					7.561 (2.678-21.349)	0.000**
Good	5	2.1	149	62.61		
Poor	17	7.14	67	28.15		
Sanitation					2.629 (1.031-6.705)	0.043*
Good	7	2.94	119	50		
Poor	15	6.3	97	40.76		
Medical control					5.526 (0.724-42.195)	0.099
Good	1	0.42	45	18.91		
Poor	21	8.82	171	71.85		
Education level					11.170 (1.474-84.673)	0.020*
High	1	0.42	75	31.51		
Low	21	8.82	141	59.24		
Income rate					10.283 (1.356-77.987)	0.024*
High	1	0.42	71	29.83		
Low	21	8.82	145	60.92		
Main job as a pig farmer					5.652 (1.287-24.825)	0.022*
No	2	0.84	78	32.77		
Yes	20	8.4	138	57.98		
Presence of *Ascaris* spp. infected pigs					0.991 (0.389-2.466)	0.984
No	14	5.88	137	57.56		
Yes	8	3.36	79	33.19		
Presence of pigsty in yard					1.244 (0.498-3.105)	0.640
No	14	5.88	148	62.18		
Yes	8	3.36	68	28.57		

** Highly significant,

* Significant, OR=Odds ratio, CI=Confident interval

**Table 5:** Risk factor of hookworm infection in traditional pig farmers in Bali.

Risk factor	Positive		Negative		OR (95% CI)	p-value
	
	Frek	%	Frek	%		
Personal hygiene					3.548 (1.704-7.391)	0.001**
Good	14	5.88	140	58.82		
Poor	22	9.24	62	26.05		
Sanitation					1.310 (0.644-2.666)	0.456
Good	17	7.14	109	45.8		
Poor	19	7.98	93	39.08		
Medical control					0.673 (0.292-1.55)	0.352
Good	9	3.78	37	15.55		
Poor	27	11.34	165	69.33		
Education level					6.225 (1.845-21.006)	0.003**
High	3	1.26	73	30.67		
Low	33	13.87	129	54.2		
Income rate					1.623 (0.701-3.59)	0.258
High	8	3.36	64	26.89		
Low	28	11.76	138	57.98		
Main job as a pig farmer					2.891 (1.15-7.268)	0.024*
No	6	2.52	74	31.09		
Yes	30	12.61	128	53.78		
Presence of hookworm infected pigs					2.102 (0.876-5.046)	0.096
No	7	2.94	68	28.57		
Yes	29	12.18	134	56.3		
Presence of pigsty in yard					1.247 (0.594-2.62)	0.56
No	23	9.66	139	58.4		
Yes	13	5.46	63	26.47		

** Highly significant,

* Significant, OR=Odds ratio, CI=Confident interval

**Table 6:** Risk factor of *Trichuris* spp. infection in traditional pig farmers in Bali.

Risk factor	Positive		Negative		OR (95% CI)	p-value
	
	Frek	%	Frek	%		
Personal hygiene					8.00 (1.658-38.598)	0.01**
Good	2	0.84	152	63.87		
Poor	8	3.36	76	31.93		
Sanitation					4.769 (0.991-22.953)	0.05*
Good	2	0.84	124	52.1		
Poor	8	3.36	104	43.7		
Medical control					2.213 (0.273-17.921)	0.457
Good	1	0.42	45	18.91		
Poor	9	3.78	183	78.15		
Education level					4.412 (0.549-35.469)	0.163
High	1	0.42	75	31.51		
Low	9	3.78	153	64.29		
Income rate					1.772 (0.367-8.559)	0.476
High	2	0.84	70	29.41		
Low	8	3.36	158	66.39		
Main job as a pig farmer					2.422 (0.279-21.025)	0.422
No	9	0.42	218	91.6		
Yes	1	3.78	10	4.2		
Presence of *Trichuris* spp. infected pigs					2.422 (0.279-21.025)	0.422
No	9	3.78	218	91.6		
Yes	1	0.42	10	4.2		
Presence of pigsty in yard					1.444 (0.395-5.277)	0.578
No	6	2.52	156	69.33		
Yes	4	1.68	72	30.25		

** Highly significant,

* Significant. OR=Odds ratio, CI=Confident interval

This study found that the state of household sanitation was a risk factor for STH infections among traditional pig farmers in Bali. In addition, data in Table-3 demonstrate that inadequate home sanitation was a risk factor for STH infection among infected pig farmers and increased the likelihood of infection by 1.914 times compared to other individuals with good sanitary practices (OR: 1.914; CI: 1.024-3.576; p=0.042). Poor home sanitation was also associated with an increased risk of infection with *Ascaris* spp. (OR: 2.629; CI: 1.031-6.705; p=0.043) and *Trichuris* spp. (OR: 4.769; CI: 0.991–22.953; p=0.05) among traditional pig farmers (Table-6), but not with hookworm infection (p>0.05) (Table-5). Furthermore, these results are consistent with those of previous studies, which indicated that STH infections were significantly related to the economic situation of the household and environmental sanitation [8,29,71]. These findings parallel another study that revealed that poor sanitation was a risk factor for schoolchildren in Rejosari village, Karangawen Demak, Indonesia, who were infected with hookworms [72].

Subsequently, the education level of traditional pig farmers was found to be a highly significant risk factor for STH worm infection among traditional pig farmers in Bali (OR: 7.579; 95% CI: 2.621-21.915; p=0.00) (Table-3). In addition, it was previously known that pig farmers with a higher level of education were less likely to be infected with STH worms. In comparison, those with a lower level of education were more likely to be infected by *Ascaris* spp. (OR: 11.170; CI: 1.474-84.673; p=0.02) and hookworm (OR: 6.225; CI: 1.845-21.006; p=0.003) (Table-5), but not with *Trichuris* spp. (p>0.05) (Table-6). This is likely because an increased level of education is associated with improved knowledge related to disease prevention, including STH infections. These results are consistent with a previous study, which showed a higher incidence of worm infection among schoolchildren whose parents had a lower education level than those whose parents had a higher level of education [73]. A similar observation was made among vegetable farmers at Barito Kuala District in Kalimantan [74].

Household income is another risk factor associated with a significant increase in the incidence of STH infection among traditional pig farmers (OR: 2.447; 95% CI: 1.122-5.338; p=0.025) (Table-3). In addition, it was discovered that there was a 2.447 times higher risk of contracting STH infections among pig farmers who earn < 2 million Rupiah per month than among those who earn more. However, household income was only a risk factor for infections with *Ascaris* spp. (OR: 5.652; CI: 1.287-24.825; p=0.022) (Table-4). Ngui *et al*. [56] demonstrated the diversity of STH infections in subgroups based on poverty and poor personal hygiene, which are significant risk factors for infection. Risk profile analyses also indicated that the Orang Kuala subgroup had a low infection rate due to the developed infrastructure and higher standard of living. Anuar *et al*. [75] found that ascariasis was significantly associated with children under the age of 15 and low household income.

It was also discovered that having a primary occupation of traditional pig farmers was a risk factor for STH infections (OR: 2.95; CI: 1.356-6.415; p=0.006) (Table-3). The results showed that pig farmers were 2.95 times more likely to contract an STH infection than respondents with a different primary occupation. Therefore, it was implied that the primary occupation of a traditional pig farmer is associated with an increased risk of infection with *Ascaris* spp. (OR: 5.652; CI: 1.287-24.825; p=0.022) and hookworm infection (OR: 2.891; CI: 1.15-7.268; p=0.024) (Tables-4 and 5). It is noteworthy that the pig rearing system in Bali consisted of traditional and simple practices, such as tying ropes to stakes. In addition, roofs were not provided to protect animals from the sun and rain. Therefore, pigs and buffalos were frequently soaked in mud after it rained [76]. This compromised the hygiene of traditional pig farms and increased the risk of contracting zoonotic diseases.

## Conclusion

This study found a high prevalence of STH infections among traditional pig farmers in Bali, with a 21.8% infection rate. This prevalence was further delineated as consisting of 15.1% hookworm infection, 9.2% infection by *Ascaris* spp., and 4.2% infection by *Trichuris* spp., with some coinfections. The risk factors associated with infections of STH and *Ascaris* spp. were personal hygiene, home sanitation, education level, household income, and having a primary occupation of a traditional pig farmer. In contrast, personal hygiene, education level, and primary occupation were the only risk factors for hookworm infection, while personal hygiene and home sanitation were the risk factors associated with *Trichuris* spp. infection.

## Authors’ Contributions

KKA: Designed the study, performed the laboratory work, analyzed the data, and wrote the manuscript. IMAW: Designed the study, analyzed the data, and wrote the manuscript. IMS: Designed the study, analyzed the data, and wrote the manuscript. MS: Designed the study, performed the laboratory work, analyzed the data, and wrote the manuscript. NSD: Designed the study, analyzed the data, and wrote the manuscript. All authors read and approved the final manuscript.
